# When Medical News Comes from Press Releases—A Case Study of Pancreatic Cancer and Processed Meat

**DOI:** 10.1371/journal.pone.0127848

**Published:** 2015-06-17

**Authors:** Joseph W. Taylor, Marie Long, Elizabeth Ashley, Alex Denning, Beatrice Gout, Kayleigh Hansen, Thomas Huws, Leifa Jennings, Sinead Quinn, Patrick Sarkies, Alex Wojtowicz, Philip M. Newton

**Affiliations:** College of Medicine, Swansea University, Singleton Park, Swansea SA2 8PP Wales, United Kingdom; Garvan Institute of Medical Research, AUSTRALIA

## Abstract

The media have a key role in communicating advances in medicine to the general public, yet the accuracy of medical journalism is an under-researched area. This project adapted an established monitoring instrument to analyse all identified news reports (n = 312) on a single medical research paper: a meta-analysis published in the British Journal of Cancer which showed a modest link between processed meat consumption and pancreatic cancer. Our most significant finding was that three sources (the journal press release, a story on the BBC News website and a story appearing on the ‘NHS Choices’ website) appeared to account for the content of over 85% of the news stories which covered the meta analysis, with many of them being verbatim or moderately edited copies and most not citing their source. The quality of these 3 primary sources varied from excellent (NHS Choices, 10 of 11 criteria addressed) to weak (journal press release, 5 of 11 criteria addressed), and this variance was reflected in the accuracy of stories derived from them. Some of the methods used in the original meta-analysis, and a proposed mechanistic explanation for the findings, were challenged in a subsequent commentary also published in the British Journal of Cancer, but this discourse was poorly reflected in the media coverage of the story.

## Introduction

Accurate media coverage is essential to maintain public trust in science and medicine. The public mainly learn about advances in science and medicine through the mainstream media (newspapers, TV, radio) [1], although with the advent of the internet the distinction between media formats has blurred, with most people obtaining news, on a daily basis, through multiple online sources including social media [2]. There has also been an increasing ‘incidental’ exposure to news online [3]. There is considerable debate about the accuracy of medical journalism but very few actual data to inform this debate, especially in the United Kingdom where the current study originates. Studies from the USA and Australia [4,5] have developed a set of criteria for use when evaluating news stories about new medical treatments. These criteria include an assessment of whether news stories accurately represent the quality of the data and the novelty of the approach, whether they accurately quantify the benefits and harms of new treatments, and whether or not they derive their information from sources beyond the journal press release. Many news stories from these countries perform poorly when assessed against the aforementioned criteria [4,5]. Previous studies have also raised concern about the accuracy of biomedical press releases [6], and the amount of ‘spin’ contained therein [7]. It has been demonstrated that scientific studies which are the subject of press releases are much more likely to feature in mainstream news reports [8], which are then often based heavily on the content of that press release, regardless of whether the content is accurate or flawed [9]. From the journalists perspective, these findings come in the context of increasing commercial and time pressures and, in many countries, a reduction in the number of specialist science reporters and training in science journalism [10]. These developments are associated with the emergence of ‘PR-led news’ and a perception, by scientists, that the quality of science news reporting is poor [11].

Diet and dietary advice are particular areas where research has found problems in the media coverage of medicine and science. A study conducted in the UK found that less than a third of all dietary advice given in UK newspapers (across one week) was supported by levels of evidence that were considered satisfactory [12]. Diet is also one area where ‘official’ (i.e. government or medically endorsed) advice may undergo frequent modification or even a complete U-turn as new evidence emerges (e.g. as in the case of the health benefits/risks of margarine [13]. Studies in Australia found that dietary advice in the media often comes from a commercial background [14] and may appear to contradict and ‘overpower’ the aforementioned official advice (e.g. see ‘The MasterChef Effect [15]). These factors may all combine to undermine public trust in the dietary advice they receive, from any source, and underscore the importance of rigorous, unbiased reporting.

We conducted a case study of one scientific publication; a meta-analysis examining whether there is a relationship between consumption of red/processed meats and the development of pancreatic cancer [16]. Pancreatic cancer is a relatively rare cancer and does not feature often in the media, although the deaths of high profile celebrities may cause a spike in coverage [17]. Coverage of cancer in the media has been a source of concern for many years, with studies showing an emphasis on ‘scary statistics’ [18] and a heavy, though often unreliable, reliance on press releases [19].

The meta-analysis studied here, by Larsson and Wolk from the Karolinska Institute in Sweden, analysed 11 studies and found, from 7 of them, a small but significant relationship between the self-reported consumption of processed meat and the risk of developing pancreatic cancer. Specifically, the self-reported daily consumption of 50g of processed meat was associated with a 19% increase in risk. In the discussion, the authors speculated on possible mechanistic explanations for the association they observed, including a putative relationship between the consumption of nitrites/nitrates and the risk of developing cancer [16]. The Larsson and Wolk paper provoked discussion within the biomedical community; six months after publishing the initial study, the British Journal of Cancer also published a commentary from David Lightsey [20], representing the organisations ‘National Council Against Health Fraud’ and ‘Quackwatch’. Lightsey expressed concern at what he considered to be limitations with the methodology used by Larsson and Wolk and their interpretation of the findings, in particular the link between nitrites/nitrates and cancer. Lightsey cited a study by Hord and co-workers [21], and a subsequent commentary by Katan [22], which both argued that the toxicity of nitrites is, at best, controversial, or at worst, significantly overstated. Subsequently, a further large study into processed meat consumption and pancreatic cancer risk found no relationship, appearing to directly contradict the findings of Larsson and Wolk [23]. We adapted the aforementioned criteria for the analysis of medical news stories [4] so that they specifically addressed issues relevant to media coverage of the Larsson and Wolk meta-analysis. These criteria were then used to analyse 312 distinct news stories reporting on the original Larsson and Wolk meta-analysis [16].

## Methods

News stories were identified by internet searches using Google, Google News, and the Nexis database. Searches were carried out between November 2012 and September 2013. Search terms were derived using basic information from the meta-analysis (see terms below). If one news story then identified further relevant stories (e.g. as sources or links) then these were also included. Search terms were entered into Google and the search results analysed page-by-page until the researcher was satisfied that no further stories could be found (by searching through the results until no relevant stories had been returned for at least 5 screen pages).

Phrases used to search Google/Nexis:

- 'british journal cancer processed meat cancer' (this search alone produced ‘about 290000 results’, in which we found 150 news stories about the Larsson and Wolk meta-analysis)
- “an extra 50g of processed meat every day increases a person’s risk by 19%”
- ‘Larsson Wolk pancreatic cancer’
- “11 trials and 6,643 patients with pancreatic cancer”
- ‘Karolinska Institute pancreatic cancer’
- ‘Lightsey pancreatic cancer’
- ‘Eating one sausage daily could raise the risk of pancreatic cancer by fifth, found a research’
- “meat ‘linked to pancreas cancer’”
- “The sample sizes ranged from 17,633 to 1,102,308, and the number of pancreatic cancer cases that occurred in the studies varied from 57 to 3,751”

The last two phrases were used specifically to search for stories which reproduced a story first seen on the NHS choices site, once it became apparent that many had done so.

News stories had to meet the following criteria to be included in the analysis:

- Focus was specifically the Larsson and Wolk meta-analysis, interpretation and analysis of findings
- English language
- Accessible online
- Written (rather than audio or video)
- Not a forum/social media (e.g. Facebook/Twitter) posting or other ‘chat site’

Stories from ‘conventional’ news outlets were included, as well as the websites of trade journals, health sites, blogs and any other site which might reasonably be considered to be ‘reporting’ on Larsson and Wolk. The full list of 312 stories analysed is given in S1 File. Once an article was deemed fit for inclusion in the study, it was analysed according to the criteria shown below.

Criteria were developed by two authors (JT and PN) using the established Health News Review/Media Doctor criteria as the starting point [4,5,24]. These were refined through discussion between the two authors, using an iterative process; 14 news stories were analysed using the original criteria, with the original Larsson and Wolk study [16] and the Lightsey commentary [20] used as context. It was clear from a detailed reading of these stories that some additional criteria would be required to address specific points relevant to the Larsson and Wolk study, particularly some of those raised by the Lightsey commentary and which also featured in the ‘NHS Choices story’ (e.g. the contribution of multiple factors to the risk of pancreatic cancer; issues with ‘recall’ and epidemiological data generally; disagreement regarding the mechanistic explanation offered by Larsson and Wolk). It was also agreed that some criteria should remain but additional detail should be added to make each criterion more specific (e.g. the Health News criterion for ‘does the story adequately quantify benefits was revised to ‘adequately quantify risk’, in keeping with the original intent of the criterion as defined by Media Doctor). Finally some of the original criteria were deemed unsuitable (e.g. ‘adequately discuss cost, establish true novelty of the idea.). The same sample of stories was then reanalysed as part of a slightly larger pool of 33 stories in total. Following further discussion between the two researchers, an additional ‘sub’ criterion was added for use when assessing if a story was derived largely or completely from a secondary source; whether this was made clear in the story (e.g. through the inclusion of a link to the original source).These then formed the final criteria used for all news stories (see below).

We did not consider it possible or useful to try and objectively make a distinction between the individual criteria regarding their relative importance for public understanding (see also discussion). For this reason we have not calculated ‘scores’ for individual stories, or groups of stories (e,g. groups according to media type). The inclusion of the points raised by Lightsey does not represent an endorsement, or otherwise, of his views (or indeed those of Larsson and Wolk). However, the fact that the same journal chose to publish both the original study and the subsequent critical commentary is indicative of the fact that the journal considered both sets of views to have approximately equal validity. Two of the points raised by Lightsey were also included in the story on the NHS Choices site (published 6 months earlier).

Stories which satisfied a criterion were scored ‘1’, those which did not were scored ‘0’, thus a higher score was reflective of a ‘better’ story. Based upon the original analysis methods developed by the ‘Media Doctor’ system, each news story was independently analysed against the criteria by a minimum of two raters from the study team, using the guidance provided above, with any scoring conflicts resolved either through discussion between the raters or by the use of an additional rater [4,5,25].

**Criteria for analysis (stories scored as ‘1’ or ‘0’, where 1 is the desired outcome for a ‘good’ story).** Every story was assessed against every criterion. For each criterion, the analysis proceeded to ask does the story;

1 **adequately quantify the increased risk of pancreatic cancer?** Pancreatic cancer is rare; the lifetime risk is approximately 1 in 77 for men and 1 in 79 for women, so a 19% increase in risk is actually very little. To satisfy this criterion, a news story had to report—or make some attempt to explain—the absolute risk of getting pancreatic cancer.
2 **grasp the quality of evidence?** The Health News Review [4] use this criterion to identify news stories which report on findings from conferences, prior to peer review and publication, or from anecdotal reports, or from small, poorly controlled studies. In this case, the quality of evidence was good (meta analysis) and the caveats were largely described in the original paper. Satisfying this criterion required merely pointing out that Larsson and Wolk had analysed 11 previous studies, which had involved many thousands of cases of pancreatic cancer e.g. is a meta-analysis of 11 studies, and/or explain what that means
3 **not represent general 'disease mongering'?** Potential examples could include—sensationalist headline, omission of key facts to increase the perception of risk, generalise risk to all cancers. A failure to satisfy other criteria could also result in a perception of disease mongering (e.g. failure to report absolute risk) but those would not result in a zero score for this criterion (i.e. they should not be double counted).
4 **use independent sources?** i.e. not from the author(s), or the journal where the meta-analysis was published (British Journal of Cancer), or Cancer Research UK (which owns the British Journal of Cancer, according to the ‘Aims and Scope’ of the journal [26]).
5 **not represent a potential, unacknowledged conflict of interest?** e.g. is from an anti/pro-meat eating/industry publication.
6 **discuss/acknowledge that the Larsson and Wolk study did not control for variables such as body-mass index and a history of diabetes?** (From Lightsey [20])
7 **discuss/acknowledge difficulties with using recall and epidemiological data to make accurate predictions about risk?** (From Lightsey [20])
8 **discuss the limitations of linking the Larsson and Wolk results to a hypothesis about nitrites**? Larsson and Wolk presented a link between nitrites and pancreatic cancer as a ‘plausible’ mechanistic explanation for their findings. This was challenged by Lightsey [20]. To satisfy this criterion, a news story had to at least acknowledge the controversy.
9 **draw in other findings and present a bigger picture**? Did the article put the news story into context of other findings on a similar topic—e.g. other studies looking at pancreatic cancer, or the effects of processed/red meats on other cancers. This criterion is derived from the Health News Review criterion suggesting stories (about new medical treatments) should ‘establish the true novelty of the treatment’ and ‘compare the new treatment with existing alternatives’.
10a **contain a significant amount of original journalism and not represent a cut-and paste from another source?** This related directly to the journalism, i.e. was there sufficient additional material to say that the news story is more than a simple copy from another source. Getting a ‘1’ for this did not necessarily mean that the rater thought journalist had read the paper itself. Some stories appeared to be copied directly from another source and then edited down, perhaps with the odd extra line/statement. These would also score ‘0’.
10b If ‘0’ to 10a, is the source of the material cited?
11 **adequately credit Larsson and Wolk**? To satisfy this criterion, the story had to give sufficient information for readers to understand who had conducted the study and where it was published. (e.g. link to/describe source of paper).

In addition, records were also made of the length of the news story (words) and the date published (where available).The length of individual stories was determined by copying the text of the story into Microsoft Word and using the ‘word count’ feature. In a small number of cases, the ability to copy text from a website was restricted and so words were counted directly off the screen.

## Results

312 news stories were identified, including reports from major national and local newspapers and other news sources around the world, as well as health sites and independent blogs (see S1 File for the full list). The combined performance of the stories against the assessment criteria is shown in Table 1.

**Table 1:** Performance of news stories against the analysis criteria.

Criterion	% of all stories meeting criterion (n = 312)
1. Adequately quantify the increased risk of pancreatic cancer?	70.8
2. Grasp the quality of evidence?	90.4
3. Represent general disease mongering?	85.3
4. Use independent sources?	64.7
5. Represent a potential, unacknowledged conflict of interest	93.6
6. Discuss/acknowledge that the Larsson and Wolk study did not control for variables such as body-mass index and a history of diabetes	50.6
7. Discuss/acknowledge difficulties with using recall and epidemiological data to make accurate predictions about risk	49.4
8. Discuss the limitations of linking the Larsson and Wolk results to a hypothesis about nitrites.	0.6
9. Draw in other findings and present a bigger picture	72.4
10. Contain a significant amount of original journalism and not represent a cut-and paste from another source.	14.4
10b If ‘0’ to 10a, is the source of the material cited?	43.8*^1^
11. Adequately credit Larsson and Wolk	84.3

Performance of all stories is shown, apart from for criterion 10b which was only analysed where a story failed criterion 10.

*1 Only of stories which scored ‘0’ for criterion 10 (n = 266)

Perhaps the most striking statistic from Table 1 is that 85.6% of all stories were derived wholly or largely from a secondary source. The performance of those remaining stories which appeared to be largely ‘original’ (n = 46) is shown in Table 2. Here it can be seen that the points raised by David Lightsey are covered in less than a third of those stories, while the presentation of absolute (rather than relative) pancreatic cancer risk is reported by less than 40% of stories. All other criteria are met by more than half the stories analysed.

**Table 2:** Performance of those news stories which were considered to be largely ‘original’ journalism (i.e. satisfied criterion 10).

Criterion	% of ‘primary’ stories meeting criterion (n = 46)
1. Adequately quantify the increased risk of pancreatic cancer?	39.1
2. Grasp the quality of evidence?	78.3
3. Represent general disease mongering?	67.4
4. Use independent sources?	56.5
5. Represent a potential, unacknowledged conflict of interest	78.3
6. Discuss/acknowledge that the Larsson and Wolk study did not control for variables such as body-mass index and a history of diabetes	37.0
7. Discuss/acknowledge difficulties with using recall and epidemiological data to make accurate predictions about risk	30.4
8. Discuss the limitations of linking the Larsson and Wolk results to a hypothesis about nitrites.	2.2
9. Draw in other findings and present a bigger picture	69.6
10. Contain a significant amount of original journalism and not represent a cut-and paste from another source.	100
10b If ‘0’ to 10a, is the source of the material cited?	-
11. Adequately credit Larsson and Wolk	73.9

Further analysis of those 266 stories (85.2%) which did not meet criterion 10 (‘Contains a significant amount of original journalism and not represent a cut-and paste from another source’) revealed that all but one were derived largely or wholly from 1 of 3 sources. These sources were journal press release, BBC (British Broadcasting Corporation) News and NHS Choices. The host institute for the Larsson and Wolk study also issued a press release, but this appeared to be derived from the journal press release. A comparison of the performance of these stories with the performance of their source story is shown in Table 3. The general pattern revealed is that those criteria which were satisfactorily addressed in the source were largely also addressed in the stories derived from that source, while those categories which the source did not address were largely not satisfied in the derived stories. For the NHS choices-derived stories, this was largely a direct relationship as the stories were all verbatim copies of each other. However, for the journal press release and BBC News sourced stories the relationship was less clear. Of these, 88 were derived largely from the press release, including 27 which appeared to be derived from stories that had themselves been derived from the press release. 40 stories were from an article on the BBC news website, or combinations of other secondary/tertiary sources including a significant contribution from the BBC. Further analysis of these press release/BBC-derived stories revealed that 90% of them were shorter than the source from which they were derived (79/88 for press release-derived stories, mean word length of 323 +/- 23 (standard error of the mean) compared to 487 words for press release, 35/39 for BBC-derived stories, one story no longer available when word count analysis performed) mean of 372 +/- 26 compared to 534 words for original BBC story). This indicates that most of the stories derived from these sources were edited down from the original source prior to publication. To determine whether this editing was related to the number of criteria successfully addressed by the story, we calculated a Pearson correlation coefficient for these stories. This showed a highly significant, positive correlation between the length of a story (in words) and the number of categories it successfully addressed (r = 0.6371, n = 127, P<0.0001). Contained within the press-release derived stories were most of the major news organisations in the United Kingdom (where the study was originally published) and many international news organisations. No UK mainstream news organisations were found to have addressed criterion 10 (see S1 File for full list of stories).

**Table 3:** Performance of those news stories which failed to meet criterion 10, compared against their source story.

Criterion	Stories not meeting criterion 10, and their source					
	Press release (n = 88)		BBC*^3^ (n = 40)		NHS Choices*^1^ (n = 137)	
	Stories	Source	Stories	Source	Stories	Source
1. Adequately quantify the increased risk of pancreatic cancer?	45.5	1	65.0	1	100	1
2. Grasp the quality of evidence?	84.1	1	85.0	1	100	1
3. Represent general disease mongering?	69.3	1	90.0	1	100	1
4. Use independent sources?	26.1	0	37.5	1	100	1
5. Represent a potential, unacknowledged conflict of interest	93.2	0*^2^	90.0	1	100	1
6. Discuss/acknowledge that the Larsson and Wolk study did not control for variables such as body-mass index and a history of diabetes	2.3	0	2.5	0	100	1
7. Discuss/acknowledge difficulties with using recall and epidemiological data to make accurate predictions about risk	1.1	0	2.5	0	100	1
8. Discuss the limitations of linking the Larsson and Wolk results to a hypothesis about nitrites.	0.0	0	2.5	0	0	0
9. Draw in other findings and present a bigger picture	35.2	0	62.5	1	100	1
10. Contain a significant amount of original journalism and not represent a cut-and paste from another source.	0.0	-	0.0	1	-	-
10b If ‘0’ to 10a, is the source of the material cited?	47.7	-	85.0	-	30.7*^4^	-
11. Adequately credit Larsson and Wolk	65.9	1	82.5	1	100	1

Values shown for ‘stories’ are percentages of the total number of stories derived from that source. In general, categories which were represented in the source (press release, BBC News or NHS Choices) were addressed in the stories derived from that source.

*1 137 identical stories were identified, all apparently derived from NHS Choices (see text)

*2 The press release scored zero here merely because the charity responsible for it also own the journal in which the original paper is published.

*3 This includes some stories which were combinations of more than one secondary source, but for which the BBC was a significant contributor

*4 Only stories featuring on the websites of medical centres cited the source (as NHS Choices). No newspapers cited the source (see S1 File)

**Table 4** shows a comparison between the press release/BBC-derived stories and those which appeared to be original. Although we did not consider it valid to conduct a detailed statistical comparison of the performance of these groups (see methods), it can be seen that the press release-derived stories fared poorly compared to the other groups, using a simple measure of how many stories satisfied each criterion.

**Table 4:** Performance of those news stories which failed to meet criterion 10, compared against those stories which did.

Criterion	Primary (n = 46)	Press release (n = 88)	BBC (n = 40)
1. Adequately quantify the increased risk of pancreatic cancer?	39.1	45.5	***65*.*0***
2. Grasp the quality of evidence?	78.3	84.1	***85*.*0***
3. Represent general disease mongering?	67.4	69.3	***90*.*0***
4. Use independent sources?	***56*.*5***	26.1	37.5
5. Represent a potential, unacknowledged conflict of interest	78.3	***93*.*2***	90.0
6. Discuss/acknowledge that the Larsson and Wolk study did not control for variables such as body-mass index and a history of diabetes	***37*.*0***	2.3	2.5
7. Discuss/acknowledge difficulties with using recall and epidemiological data to make accurate predictions about risk	***30*.*4***	1.1	2.5
8. Discuss the limitations of linking the Larsson and Wolk results to a hypothesis about nitrites.	2.2	0.0	***2*.*5***
9. Draw in other findings and present a bigger picture	***69*.*6***	35.2	62.5
10. Contain a significant amount of original journalism and not represent a cut-and paste from another source.	***100***	0.0	0.0
11. Adequately credit Larsson and Wolk	73.9	65.9	***82*.*5***

Stories are organised according to the source from which they were derived (excluding those derived from the ‘NHS Choices’ story) and are compared to those which met criterion 10 (primary). Simple percentages of stories meeting the criterion are given, and the story type ranking highest of the three is shown in italicised bold.

As described above, 137 stories were identical verbatim copies of each other, appearing on the NHS Choices (United Kingdom National Health Service) website, and 91 local newspapers in the UK and Ireland, mostly owned by the Johnson Media group and the Newsquest Media group. The story also appeared in the news sections of 43 primary care centres in the UK. According to the NHS Choices Website (http://www.nhs.uk/news/pages/about-behind-the-headlines.aspx), articles appearing there are written by the company ‘Bazian’ and then contracted out to primary care centres, thus it seems likely that the NHS Choices/Bazian site is the source for these stories, including those appearing in the aforementioned local newspapers. All 137 of these stories were 1667 words long and met 10 of the 11 criteria.

A date of publication was given by 65.4% of stories, and of these 87% were published within one week of the press release. No news stories were found which cited the Lightsey commentary, which was published 6 months after the press release.

## Discussion

The present study demonstrates the paucity of primary sources in the media reporting of a meta-analysis on pancreatic cancer and processed meat. Of the 312 news stories identified, only 14.4% went beyond a secondary source. Previous studies in this area, analysing individual news stories (largely from the USA), have found that approximately one third of them are derived from journal press releases [27]. The current approach, analysing all the news stories that we could find about an individual scientific study, had a similar result; approximately one third were derived largely or wholly from the press release. However, in this study, two other ‘primary’ sources were identified. These were then the basis for a further 177 (57%) of stories found. In keeping with previous studies in this area, the press release did not score highly, meeting only 5 out of the 9 criteria. Neither the press release or the BBC news story addressed any of the alleged limitations raised by David Lightsey and published in the same journal as the original study (although 6 months later) [20]. This pattern was reflected in the 128 news stories which were derived from these two sources, which largely scored well for the categories covered in the press release, but not for the concerns of Lightsey [20]. Previous studies have raised concerns about accuracy and ‘spin’ in scientific press releases [6,7,28], which are highly likely to feature in the majority of resulting news stories [9]. Further still, news stories often introduce inaccuracies of their own [19].

A finding of inaccurate media coverage of a science story relating to dietary advice is unfortunately not new, although the area remains under researched [12]. Previous studies focused exclusively on UK media have reported similar findings of exaggerated and inaccurate claims [12,29] which, when added to the need for ‘official’ advice to change as new evidence becomes available (e.g. [13]) all give grounds for a public mistrust in the way scientific dietary advice is represented in the press. We focussed on one scientific study and the 312 stories we found which reported on that study. Our findings demonstrate the potential reach of concerns regarding poor coverage, particularly those arising from an over-reliance on reproducing sources which themselves contained weaknesses that then appeared in 128 distinct news stories around the world, including many multinational media organisations, in most cases without identifying the source.

The causes of these concerns are varied, and there is a growing recognition that the responsibility for ensuring accurate media coverage lies with all those involved, not just journalists [30]. The present study does not attempt to lay ‘blame’ for our primary observation that the majority of news coverage on a single study was formed by reproducing secondary sources, and that most of the coverage was poor as a result. The last 10–20 years have seen significant changes in the organisation and structure of the media. There have also been changes to the means through which the media interacts with the public, who now access a considerable amount of material online, in a participatory, interactive way, and without loyalty to a single source [2]. There have also been significant changes in the way the media interacts with scientists and, worldwide, a reduction in the numbers and training of specialist science journalists and an increase in the time available to produce news stories. This leads to a reliance on journal and institutional press releases [10,11,27] and a much greater representation in the media of science publications that have been accompanied by a press release [8]. This produces an obvious ‘weak link’ in the governance of science journalism – a flawed press release will lead to flawed media coverage [6,9]. A recent study of press releases from major universities in the UK found that 40% of them contained ‘exaggeration’, such as the inference of a causal link between correlated data, or an inference of effects in humans from data derived using animal studies. These exaggerations were then reproduced in the majority of news stories analysed [28]. The onus then is on all in this weakened chain – scientists, press officers, journalists and editors, to present findings in a balanced, critical and comprehensive manner. The directional flow of information along this chain places extra responsibility on those at the beginning; scientists. Scientific findings are, by their very nature, often extremely complex and esoteric. In the UK, organisations such as the British Science Association and the major research funding bodies now offer media training courses for scientists, while organisations such as the Science Media Centre (UK), Health News Review (USA) [4] and Media Doctor (Australia) [24] have produced independent guidelines to help journalists and media organisations ensure that common issues are addressed in coverage of science. There have also been proposals for similar guidelines to be created for the writing of press releases [30] while others have suggested that simple increases in transparency, such as naming the authors of press releases and publishing them alongside the original scientific paper, would increases accountability and allow readers to make up their own minds [31].

One common disconnect between science and the media coverage of it relates to the issue of ‘balance’. Many broadcasters are committed to media coverage that is ‘balanced’, a notable example being the UK BBC which is funded by the UK taxpayer and has strict rules on impartiality [32]. Unfortunately one suggested outcome of a rigid commitment to impartiality and ‘balance’ has been a reliance on presenting a balance of views, rather than a balance of evidence. Recent examples include the discredited link between autism and the Measles, Mumps and Rubella (MMR) vaccine (e.g. see [33]) and also stories on climate change (e.g. see [34]).

However, the present study has also demonstrated the power of a good primary news source. The article appearing on the ‘NHS Choices’ website and in 136 other locations, was of high quality and independently addressed, in part, some of the points raised by David Lightsey. Additionally, 14.5% of stories ‘went beyond a secondary source’ altogether and, in doing so, approximately one third of them also addressed 2 of the 3 main points raised by Lightsey, again despite being published before the Lightsey commentary. We did not find any news stories which cited the Lightsey commentary and at the time of writing (January 2015) the Lightsey commentary has not been cited in any scientific publication (Google Scholar, ISI Web of Science). In addition to the recommendations discussed above regarding additional guidelines, transparency and media training, these findings suggest that, in the short term, one simple solution to the ‘weak link’ in the chain connecting science and the media (i.e. the press release) would be for media organisations to make greater use of independent and well researched sources such as NHS Choices, themselves identified through research such as that presented in the current study.

There are some limitations to the current study. Despite extensive searching, we cannot claim to have identified all the news stories whose focus was the Larsson and Wolk meta-analysis. Furthermore we have no data which would have allowed us to determine how many people have read the individual stories, although the list of stories analysed includes major news organisations around the world (see S1 File). We made no distinction between ‘types’ of media outlet reporting the story, the criterion being only that the story was available online in a written form – distinctions between different types of media appear to be ever-changing, particularly those between ‘newspapers’ and ‘online’ stories [2]. We did specifically exclude forums and obvious ‘social media’ sites such as Facebook and Twitter. These sites have important and diverse roles to play in the spread of scientific and medical (mis)information, with examples ranging from enormous increases in organ donor registration following the addition of organ donor ‘status’ to Facebook profiles [35], to the spread of potentially fatal medical misinformation about treatments for the Ebola virus via Twitter [36]. We excluded forums and social media from our study and focused on more traditional news sources as these still appear to be the means through which the public access news information (although they now often do so via online portals for these organisations) [1,2] and the use of sites such as Twitter and Facebook by medical and scientific journals does not currently appear to be widespread (e.g. see [37]). However, analyses of the spread of medical information via forums and social media will be important avenues for further research into the quality and spread of scientific news coverage.

We based our analysis on published criteria for the assessment of news stories regarding science and medicine [4,5,24], which we revised to address media coverage of a particular scientific study. However the criteria themselves will not capture every important detail from every news story. As a simple example, a story that represented a conflict of interest might, or might not, add significant extra material that changed the context in which the story was presented (and thus interpreted by some readers) and this may then have a more profound influence over how the story is presented than, say, a failure to accurately quantify risk. Alternately a story may represent a conflict of interest and yet there may be no obvious influence on the presentation of the story. For this reason we did not consider it valid to try and distinguish between the relative importance of the different analysis criteria, and thus did not attempt to compare stories by calculating scores for individual stories or groups of stories (e.g. those arising from different countries or in different types of media).This distinction would not be captured using the criteria. Interactions between the criteria and the subjective nature of many of the judgements used in the analysis (see below) are limitations of the study that could be explored by using a focused, qualitative analysis, perhaps restricted to a subset of stories.

Finally, it is difficult for a study of this nature to accurately trace the scientific content of the original study. For the 46 stories (14.7% of total) that ‘were not based largely on secondary sources’, it is not possible for us to conclude that the authors of those stories have actually read the original scientific manuscript. This raises the possibility that the number of stories whose *scientific* content was informed only by a secondary source was higher than the 266 identified (whose combined scientific *and* journalistic content appeared to be based largely on a secondary source, although we also cannot conclude with certainty that a news story which is largely based upon a secondary source has been written by a journalist who has *not* read the original Larsson and Wolk study). For example, the BBC news story contained only scientific information that is also present in the press release, with additional journalism and comments from an independent source. Similarly, some of the 46 news stories identified as ‘primary’ still use the BBC as a source for some of their content but added enough original journalistic content to satisfy criterion 10; thus the reach of the BBC story was greater than the 40 stories which are largely based upon it. This last issue is part of a larger limitation which affects all studies of this type – the need for subjective decision making when assessing news stories against criteria such as those used here. The impact of this limitation was minimised through various means, for example by basing our analyses on existing, established criteria [4,20] and ensuring that each decision was made by a minimum of two raters with discussion and/or referral to a third rater used to resolve any disagreement. This is the method used in the original ‘Media Doctor’ studies [4,5,25] and in other studies of this nature (e.g.[38]), although some studies use single raters (e.g.[12]) while others use single raters plus double rating of a sample, with reliability analyses used to check for accuracy (e.g, [28,39]).

In summary, we identified 312 distinct news stories, all reporting on a single meta-analysis describing a potentially increased risk for pancreatic cancer associated with processed meat consumption. Over 85% of the news stories were based on one of three primary sources, which themselves varied in quality, amplifying the net effect of any errors or weaknesses in those sources. This emphasises the need for rigour and accuracy in scientific and medical press releases, which are the indirect source of public education about advances in science and medicine.

## Supporting Information

S1 File List of News Stories Analysed. Every story analysed in the study is listed, along with a link to the webpage (where possible).
(XLSX)
